# Incidence and prevalence of *Vibrio parahaemolyticus* in seafood: a systematic review and meta-analysis

**DOI:** 10.1186/s40064-016-2115-7

**Published:** 2016-04-14

**Authors:** Olumide A Odeyemi

**Affiliations:** Ecology and Biodiversity Centre, Institute for Marine and Antarctic Studies, University of Tasmania, Launceston, Australia

**Keywords:** Seafood safety and quality, Prevalence, Reservoir, *V. parahaemolyticus*, Shellfish

## Abstract

*Vibrio parahaemolyticus* is an important seafood borne human pathogen worldwide due to it occurrence, prevalence and ability to cause gastrointestinal infections. This current study aim at investigating the incidence and prevalence of *V. parahaemolyticus* in seafood using systematic review-meta-analysis by exploring heterogeneity among primary studies. A comprehensive systematic review and meta-analysis of peer reviewed primary studies reported between 2003 and 2015 for the occurrence and prevalence of *V. parahaemolyticus* in seafood was conducted using “isolation”, “detection”, “prevalence”, “incidence”, “occurrence” or “enumeration” and *V. parahaemolyticus* as search algorithms in Web of Science (Science Direct) and ProQuest of electronic bibliographic databases. Data extracted from the primary studies were then analyzed with fixed effect meta-analysis model for effect rate to explore heterogeneity between the primary studies. Publication bias was evaluated using funnel plot. A total of 10,819 articles were retrieved from the data bases of which 48 studies met inclusion criteria. *V. parahaemolyticus* could only be isolated from 2761 (47.5 %) samples of 5811 seafood investigated. The result of this study shows that incidence of *V. parahaemolyticus* was more prevalent in oysters with overall prevalence rate of 63.4 % (95 % CI 0.592–0.674) than other seafood. Overall prevalence rate of clams was 52.9 % (95 % CI 0.490–0.568); fish 51.0 % (95 % CI 0.476–0.544); shrimps 48.3 % (95 % CI 0.454–0.512) and mussels, scallop and periwinkle: 28.0 % (95 % CI 0.255–0.307). High heterogeneity (p value <0.001; *I*^2^ = 95.291) was observed mussel compared to oysters (*I*^2^ = 91.024). It could be observed from this study that oysters harbor *V. parahaemolyticus* based on the prevalence rate than other seafood investigated. The occurrence and prevalence of *V. parahaemolyticus* is of public health importance, hence, more studies involving seafood such as mussels need to be investigated.

## Background

*Vibrio parahaemolyticus* is a non-sucrose fermenting halophilic bacterium that grows between 10 and 44 °C and optimum temperature of 35–37 °C (Zamora-Pantoja et al. 2013; Wagley et al. 2009). The first outbreak of seafood borne disease due to consumption of *V. parahaemolyticus* contaminated sardine was reported in Japan in 1950 (Levin 2006). In this outbreak, 20 people were reported dead while over 270 people were likewise hospitalized. More outbreaks involving consumption of contaminated raw or undercooked seafood like oyster has been reported in United States (Iwamoto et al. 2010; McLaughlin et al. 2005; Drake et al. 2007), China (Liu et al. 2004), Taiwan (Chiou et al. 2000), Spain (Lozano-Leon et al. 2003), Italy (Ottaviani et al. 2008), Chile (Garcia et al. 2009), Peru (Gil et al. 2007) and (Leal et al. 2008) *V. parahaemolyticus* infection is characterized with vomiting, acute abdominal pain, abdominal pain, vomiting, watery or bloody diarrhea and gastroenteritis as result of production of thermostable direct hemolysin (TDH) and TDH-related hemolysin (TRH) toxins respectively (Jahangir Alam et al. 2002; Wagley et al. 2009) with an incubation period of 4–96 h (Levin 2006) however, non-pathogenic *V. parahaemolyticus* strains do not cause any infection. Several studies have been conducted globally regarding occurrence and prevalence of total or pathogenic *V. parahaemolyticus* in seafood yet there exist variability among the studies in terms of incidence and prevalence.

Meta-analysis is a quantitative statistical summarizing techniques aimed at extracting and combining scientific results from multiple primary studies that have investigated the same research question (Gonzales-Barron et al. 2013). Meta-analysis explains possible differences in outcomes of primary studies by extracting and encoding study characteristics such as research design features, data collection procedures, type of samples and year of study (DerSimonian and Laird 1986). This involves several steps like systematic review of literatures, data extraction of both qualitative and quantitative information from relevant primary studies, selection of effect size as described from each study, estimation of overall effect size of all the primary studies, assessment of heterogeneity of studies and presentation of meta-analysis using numerical (odd ratios, fixed effects size, p values, publication bias, meta regression, and random effect) and or graphical methods forest plot, funnel plot and others (Gonzales-Barron et al. 2013). Method of data generation differs from one study to another. Hence, researchers can either perform experiment to generate data or utilize available data from previous study (primary study) without experimental work (den Besten and Zwietering 2012). It was recently that food safety researchers stated conducting meta analytical studies as most meta-analytical study are conducted only in medical and social sciences (Gonzales Barron et al. 2008; Gonzales-Barron and Butler 2011; Patil et al. 2004). Meta-analytical studies could be carried out  in food safety research in order to help answer various research questions involving prevalence  pathogens in foods, treatment interventions, predictive modelling, microbial risk assessement, food safety knowledge, attitude and practices (Xavier et al. 2014).

Currently, no meta-analysis has been conducted on estimation of overall incidence, detection and prevalence of *V. parahaemolyticus* in seafood has been carried out in order to gain insight to source(s) of reservoir for these bacterial pathogens. This study therefore aim to systematically review and summarize primary studies describing incidence and prevalence of *V. parahaemolyticus* in seafood worldwide.

## Methods

### Definition

For the purpose of this study, incidence is defined as occurrence (presence) of *V. parahaemolyticus* in seafood samples analyzed in the primary studies while prevalence (p) is the number (n) of seafood that was positive for the presence of *V. parahaemolyticus* from the total sample (N). Primary studies imply all the studies carried out by other researchers used in this study. Population of study is the type of seafood investigated in each study. Seafood considered in this study are mollusks (oysters, clams, and mussels), finfish (salmon and tuna) and crustaceans (shrimp, crab, and lobster) (Iwamoto et al. 2010). In order to achieve the aim of this study, modified methods of Preferred Reporting Items for Systematic Reviews and Meta-Analyses—PRIMA (Moher et al. 2009) and (Gonzales-Barron and Butler 2011) were used. The steps consist of systematic review of literatures, data extraction of both qualitative and quantitative information from relevant primary studies, selection of effect size as described from each study, estimation of overall effect size of all the primary studies, assessment of heterogeneity of studies and meta-analysis representation of obtained result using numerical (odd ratios, fixed effects size, p values, publication bias, meta regression, and random effect) and or graphical methods forest plot, funnel plot and others).

### Literature search, selection and relevance screening

This review was guided by a research question and problem statement. The research question was how prevalent is *V. parahaemolyticus* in seafood? While a problem statement describing the incidence and prevalence of *V. parahaemolyticus* in different seafood samples was formulated. Presence or absent of *V. parahaemolyticus* was considered as possible outcome of each primary study. Thereafter, a comprehensive literature search of electronic databases (ISI Web of science and ProQuest) and systematic review of available primary studies aimed at producing summary of relevant, quality and initial findings from such studies was carried out. The following search algorithms: “isolation” and *V. parahaemolyticus*, “detection” and *V. parahaemolyticus*, “prevalence” and *V. parahaemolyticus,* “incidence” and *V. parahaemolyticus,* “occurrence” and *V. parahaemolyticus* and “enumeration” and *V. parahaemolyticus* were used. Preliminary screening (Abstract-based relevance screening) of titles and abstracts of retrieved primary studies was carried out for eligibility and relevance to this study. Relevance of each article was screened using both inclusion and exclusion criteria. The inclusion criteria are: description of isolation method of *V. parahaemolyticus* from seafood using both conventional method (use of Thiosulphate Citrate Bile Salt agar—TCBS**)** and or molecular methods (Polymerase chain reaction—PCR). Full text and peer reviewed articles in English. The total number (population) of samples studied and number of samples that are positive for presence of *V. parahaemolyticus* clearly stated in the study. The exclusion criteria are: review articles, detection of *V. parahaemolyticus* in artificially contaminated samples, non-peer reviewed articles such as thesis, opinion articles, non-food related sources of *V. parahaemolyticus* such as clinical samples and conference abstract due to lack of access to full articles. Thereafter, full text screening of eligible primary studies were obtained from the databases. Articles that are not freely available were obtained via the service of the University of Tasmania’s library. Citations identified were retrieved and further checked for duplication using Endnote x7.1 software.

### Data extraction and assessment of quality

Based on the inclusion and exclusion criteria, first author, year of publication or study, location, type of seafood studied, microbiological methods, number of sample positive for presence of *V. parahaemolyticus* were extracted.

### Statistical analysis of extracted data

The pooled estimates of prevalence of *V. parahaemolyticus* in seafood were obtained by fixed effect meta-analysis model. The model was used to analyze combined extracted data while variation of incidence and prevalence of *V. parahaemolyticus* between the primary studies was evaluated using heterogeneity (*I*^2^). Heterogeneity of prevalence estimates between the studies was investigated using Q statistic (Bangar et al. 2014) and quantified by *I*^2^ Index (Higgins et al. 2003) as shown in below equations.

1 $$Q = \sum {\left\{ {w_{i} \left( {\beta_{i} - \beta_{w} } \right)^{2} } \right\}}$$

2 $$I^{2} = \left\{ {\left( {Q - df} \right)/Q} \right\}\%$$

where df is the degree of freedom (N − 1), *β*_*w*_ is the pooled estimate, *β*_i_ is the estimate of individual primary study. Presence of bias in the publications was determined using funnel plots (odd of presence of *V. parahaemolyticus* in the samples) of standard error. Forest plots were however used to estimate the event rate at 95 % confidence intervals. Prevalence (p) and standard error (s.e.) were calculated by the following formulae: p = n/N and s.e. = √ p (1 − p)/N: where n = number of positive samples and N = number of samples (Tadesse and Tessema 2014). Modified method of (Greig et al. 2012) was used for the assessment of risk bias. Statistical analyses was carried out using Comprehensive Meta-Analysis (CMA) software. Statistical p values (p < 0.05) were considered as statistically significant.

## Results and discussion

### Literature search

**Fig. 1:**
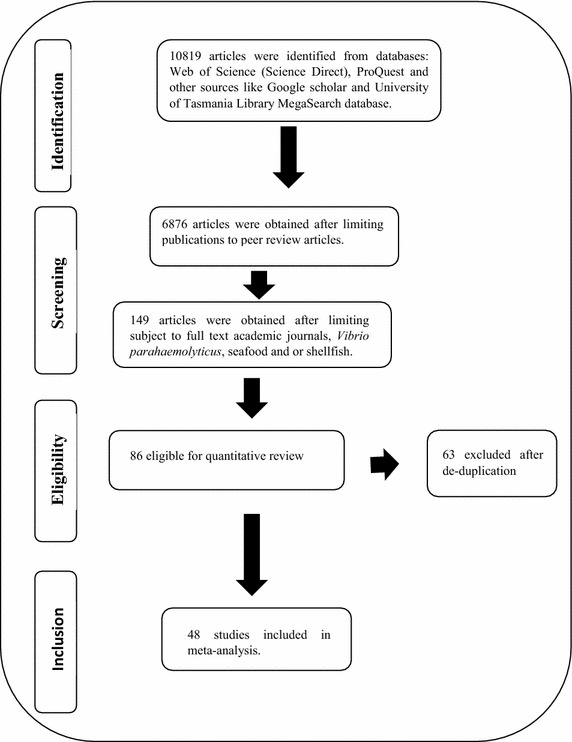
Flow diagram of selected studies included in fixed effect meta-analysis

The numbers of studies on *V. parahaemolyticus* has increased over the years. This current study is the first meta-analytical study to be carried out on incidence and prevalence of *V. parahaemolyticus* in seafood. Figure 1 shows results obtained from literature search. Literature search yielded 10,819 primary studies. However, when the source of articles was limited to peer review journals, 6876 articles were obtained. Further limiting of the subject to full text academic journals, *V. parahaemolyticus*, seafood and or shellfish, 149 articles were obtained. Abstract relevance screening of published articles reduced the study to 86 while only 63 articles remained after de-duplication. Hence, only few primary studies met the inclusion requirement of this meta-analysis. The primary studies considered in this meta-analysis described standard method for isolation and detection of *V. parahaemolyticus* from seafood samples. First author, year of publication or study, location of study, type of seafood studied, microbiological methods and number of sample positive for presence of *V. parahaemolyticus* were extracted from the following 48 primary studies: (Abd-Elghany and Sallam 2013; Amin and Salem 2012; Anjay et al. 2014; Bilung et al. 2005; Blanco-Abad et al. 2009; Chakraborty and Surendran 2008; Changchai and Saunjit 2014; Chao et al. 2009; Cook et al. 2002; Copin et al. 2012; Deepanjali et al. 2005; DePaola et al. 2003; Di Pinto et al. 2008, 2012; Dileep et al. 2003; Duan and Su 2005a, b; Eja et al. 2008; Fuenzalida et al. 2006, 2007; Han et al. 2007; Khouadja et al. 2013; Kirs et al. 2011; Koralage et al. 2012; Lee et al. 2008; Lu et al. 2006; Luan et al. 2008; Marlina et al. 2007; Miwa et al. 2006; Nakaguchi 2013; Nelapati and Krishnaiah 2010; Normanno et al. 2006; Ottaviani et al. 2005; Pal and Das 2010; Parveen et al. 2008; Paydar et al. 2013; Pereira et al. 2007; Raghunath et al. 2007; Ramos et al. 2014; Rizvi and Bej 2010; Robert-Pillot et al. 2014; Rosec et al. 2012; Schärer et al. 2011; Sobrinho Pde et al. 2011; Sobrinho et al. 2010; Sudha et al. 2012; Suffredini et al. 2014; Sun et al. 2012; Terzi et al. 2009; Vuddhakul et al. 2006; Xu et al. 2014; Yamamoto et al. 2008; Yang et al. 2008a, b; Yano et al. 2014; Zarei et al. 2012; Zhao et al. 2011; Zulkifli 2009). The outcome of this study revealed that oysters are more contaminated with this pathogen than other samples. It could be observed from this study that more studies have carried out on oyster than other samples. Oysters are eaten either raw or undercooked. This practice tend to increase the prevalence of outbreak of *V. parahaemolyticus* in oysters especially in countries like United States, China and Japan. There are limitations in meta-analysis study. Only studies that are published in English are used in this study. There could be possibility that positive results involving incidence of *V. parahaemolyticus* from other seafood are reported. This correlates with the publication bias observed in the study which involve publication of study with significant results. Additionally, primary research studies involving clinical samples were not included in this study

### Descriptive characteristics of eligible studies

As seen in Table 1, the studies were conducted and published between 2003 and 2015 from the following 24 countries: Brazil (3 studies); India (6 studies); Iran (1 study); United Kingdom (1 study); China (5 studies); Thailand (4 studies); Vietnam (1 study); Malaysia (3 studies); Indonesia (3 studies); Italy (5 studies); Japan (1 study); Chile (1 study); Egypt (2 studies); United States (3 studies); Turkey (1 study); France (3 studies); Spain (1 study); Mexico (1 study); Korea (1 study); Sri Lanka (1 study); Nigeria (1 study); Tunisia (1 study); New Zealand (1 study) and Switzerland (1 study). *V. parahaemolyticus* was isolated from 2761 (47.5 %) of 5811 mussel, scallop and periwinkle (1670) in 15 studies, oyster (951) in 17 studies, clam and cockle (830) in 18 studies,, shrimps, prawn and crab (1422) in 23 studies, fish, squid and cephalopod (998) in 20 studies of seafood investigated.

**Table 1:** Descriptive characteristic of eligible studies in meta-analysis

Sn	Sr	Ls	Yp	Ts	M	N	n	P (%)
1	Sobrinho Pde et al. (2011)	Brazil	2011	Oyster	TCBS/PCR^m^	74	74	100
2	Sudha et al. (2012)	India	2012	Finfish	TCBS/PCR	182	82	45.1
3	Zarei et al. (2012)	Iran	2012	Shrimps	TCBS/PCR	300	146	43.9
4	Wagley et al. (2009)	England	2009	Crabs	TCBS/PCR	22	22	100
5	Zhao et al. (2011)	China^a^	2011	Oyster	TCBS/PCR	80	39	48.8
				Clam	TCBS/PCR	72	46	63.8
				Scallop	TCBS/PCR	70	42	60.0
				Mussel	TCBS/PCR	76	45	59.2
6	Nakaguchi (2013)	Thailand	2013	Cockle	TCBS/PCR	109	76	69.4
				Mussel	TCBS/PCR	73	54	74.5
				Oyster	TCBS/PCR	32	27	83.3
				Clam	TCBS/PCR	86	52	60.0
		Vietnam		Fish	TCBS/PCR	16	10	62.5
				Shrimp	TCBS/PCR	18	13	73.2
				Squid	TCBS/PCR	7	2	28.6
				Crab	TCBS/PCR	5	2	40.0
		Malaysia		Fish	TCBS/PCR	11	6	54.5
				Squid	TCBS/PCR	11	6	54.5
		Indonesia		Shrimp	TCBS/PCR	37	23	62.1
				Squid	TCBS/PCR	29	4	13.8
7	Di Pinto et al. (2008)	Italy	2008	Mussel	TCBS/PCR	144	47	32.6
8	Yamamoto et al. (2008)	Thailand^b^	2008	Clams	MPN^k^/PCR	32	32	100
9	Miwa et al. (2006)	Japan	2006	Fish	MPN/PCR	30	4	13.3
				Shrimp	MPN/PCR	20	11	55.0
				Cockle	MPN/PCR	10	9	90
10	Fuenzalida et al. (2006)	Chile	2006	Mussel	TCBS/PCR	35	9	25.7
				Clam	TCBS/PCR	8	2	25
				Oyster	TCBS/PCR	5	1	20
11	Anjay et al. (2014)	India	2014	Fish	TCBS/PCR	182	140	76.9
				Prawn	TCBS/PCR	42	31	73.8
12	Abd-Elghany and Sallam (2013)	Egypt	2013	Shrimp	TCBS/PCR	40	9	22.5
				Crab	TCBS/PCR	40	8	20
				Cockle	TCBS/PCR	40	3	7.5
13	Changchai and Saunjit (2014)	Thailand	2014	Raw oysters^l^	MPN/PCR	240	219	91
14	Ramos et al. (2014)	Brazil	2014	Oyster	MPN/PCR	60	29	48.3
15	Chakraborty and Surendran (2008)	India	2008	Finfish	TCBS/MPN	12	8	66.6
				Shellfish	TCBS/MPN	25	21	84.0
				Cephalopods	TCBS/MPN	5	4	80
16	Bilung et al. (2005)	Malaysia	2005	Cockle	MPN/PCR	100	62	62
17	Rosec et al. (2012)	France	2012	Oyster	TCBS/C/PCR	60	19	31.6
				Clams/mussel	TCBS/C/PCR	9	1	11.1
18	Terzi et al. (2009)	Turkey	2009	Fish	TCBS/PCR	30	9	30
				Mussel	TCBS/PCR	60	35	58.3
19	Suffredini et al. (2014)	Italy	2014	Mussel	TCBS/PCR	75	31	41.3
				Clams	TCBS/PCR	51	22	43.1
20	Sun et al. (2012)	China	2012	Oyster	TCBS/LAMP	10	2	20
				Clam	TCBS/LAMP	16	2	12.5
21	Parveen et al. (2008)	US	2008	Oyster	TCBS/DCH/PCR	33	22	67
22	Di Pinto et al. (2012)	Italy	2012	Mussel	PCR/ELISA	195	26	13.3
23	Rizvi and Bej (2010)	Mexico	2010	Oyster	SYBR/PCR	24	14	58.3
24	Blanco-Abad et al. (2009)	Spain	2009	Mussel	TCBS/PCR	48	5	10.4
25	Marlina et al. (2007)	Indonesia	2007	Clam	RAPD/PCR	35	13	37.1
26	Luan et al. (2008)	China	2008	Shrimp	MPN/PCR	80	66	82.5
				Crab	MPN/PCR	15	14	93.3
				Clam	MPN/PCR	100	64	64
				Fish	MPN/PCR	10	10	100
				Scallop	MPN/PCR	20	11	55
27	Lu et al. (2006)	US	2006	Oyster	RAPD/PCR	13	9	69
				Mussel	RAPD/PCR	22	7	32
				Clam	RAPD/PCR	48	13	27
28	Robert-Pillot et al. (2014)	France	2014	Fish	RT/PCR	27	5	18.5
				Mussel/Scallop	RT/PCR	10	1	10
29	Zulkifli (2009)	Indonesia	2009	Cockle	C/PCR	50	25	50
30	Nelapati and Krishnaiah (2010)	India	2010	Fish	TCBS/PCR	105	69	65.7
31	Yano et al. (2014)	Thailand	2014	Shrimp	MPN/PCR	16	6	37.5
32	Duan and Su (2005a)	US	2005	Oyster	TCBS/PCR	74	31	41.9
33	Copin et al. (2012)	France	2012	Shrimp	MPN/PCR	36	28	77.8
34	Yang et al. (2008a)	China	2008	Fish	RADP/PCR	197	58	29.7
				Crab	RADP/PCR	49	22	44.9
				Shrimp	RADP/PCR	71	28	39.4
35	Ottaviani et al. (2005)	Italy	2005	Mussel	TCBS/PCR	144	35	24.3
36	Sobrinho et al. (2010)	Brazil	2010	Oyster	MPN/PCR	123	122	99.2
37	Xu et al. (2014)	China	2014	Shrimp	TCBS/PCR	273	103	37.7
38	Lee et al. (2008)	Korea	2008	Oyster	TCBS/PCR	72	48	66.7
39	Amin and Salem (2012)	Egypt	2012	Shrimp	TCBS/PCR	20	4	20
				Crab	TCBS/PCR	20	6	30
40	Koralage et al. (2012)	Sri Lanka	2012	Shrimp	TCBS/PCR	170	155	91.2
41	Schärer et al. (2011)	Switzerland	2011	Squid	TCBS/PCR	2	2	100
42	Paydar et al. (2013)	Malaysia	2013	Fish	TCBS/mPCR	27	21	77.8
				Squid	TCBS/PCR	7	4	57.1
				Cockle	TCBS/PCR	5	3	60
				Shrimp	TCBS/PCR	11	9	81.8
				Clam	TCBS/PCR	3	2	66.7
				Prawn	TCBS/PCR	7	5	71.4
				Oyster	TCBS/PCR	9	6	66.7
43	Dileep et al. (2003)	India	2003	Finfish	TCBS/PCR	18	4	22.2
				Shrimp	TCBS/PCR	10	3	30
44	Eja et al. (2008)	Nigeria	2008	Shrimp	TCBS/Biotyping	120	26	21.7
				Clam	TCBS/Biotyping	90	7	7.7
				Periwinkle	TCBS/Biotyping	98	9	9.2
45	Khouadja et al. (2013)	Tunisia	2013	Oyster	TCBS/PCR	20	2	10.0
				Mussel	TCBS/PCR	20	1	5.0
46	Kirs et al. (2011)	New Zealand	2011	Oyster	TCBS/RT/PCR	58	55	94.8
47	Normanno et al. (2006)	Italy	2006	Mussel	TCBS/API	600	47	7.83
48	Pal and Das (2010)	India	2010	Fish	TCBS/PCR	90	60	66.7

i, shucked oyster; tb, Tillamook Bay; yb, Yaquina Bay; S, Selangor; pj, Padang and Jakarta; m, use of any molecular method like specie specific genes etc, k; mpn chrom agar; a, coastal province Jiangsu; China b, eastern coast of China. Sn = study number; Sr = study reference; Ls = location of study; Yp = year of publication; Ts = type of seafood; M = microbiological method(s); N = total sample; n = number of positive samples

### Meta-analysis of prevalence of *V. parahaemolyticus* in mussel, scallop, and periwinkle

**Fig. 2:**
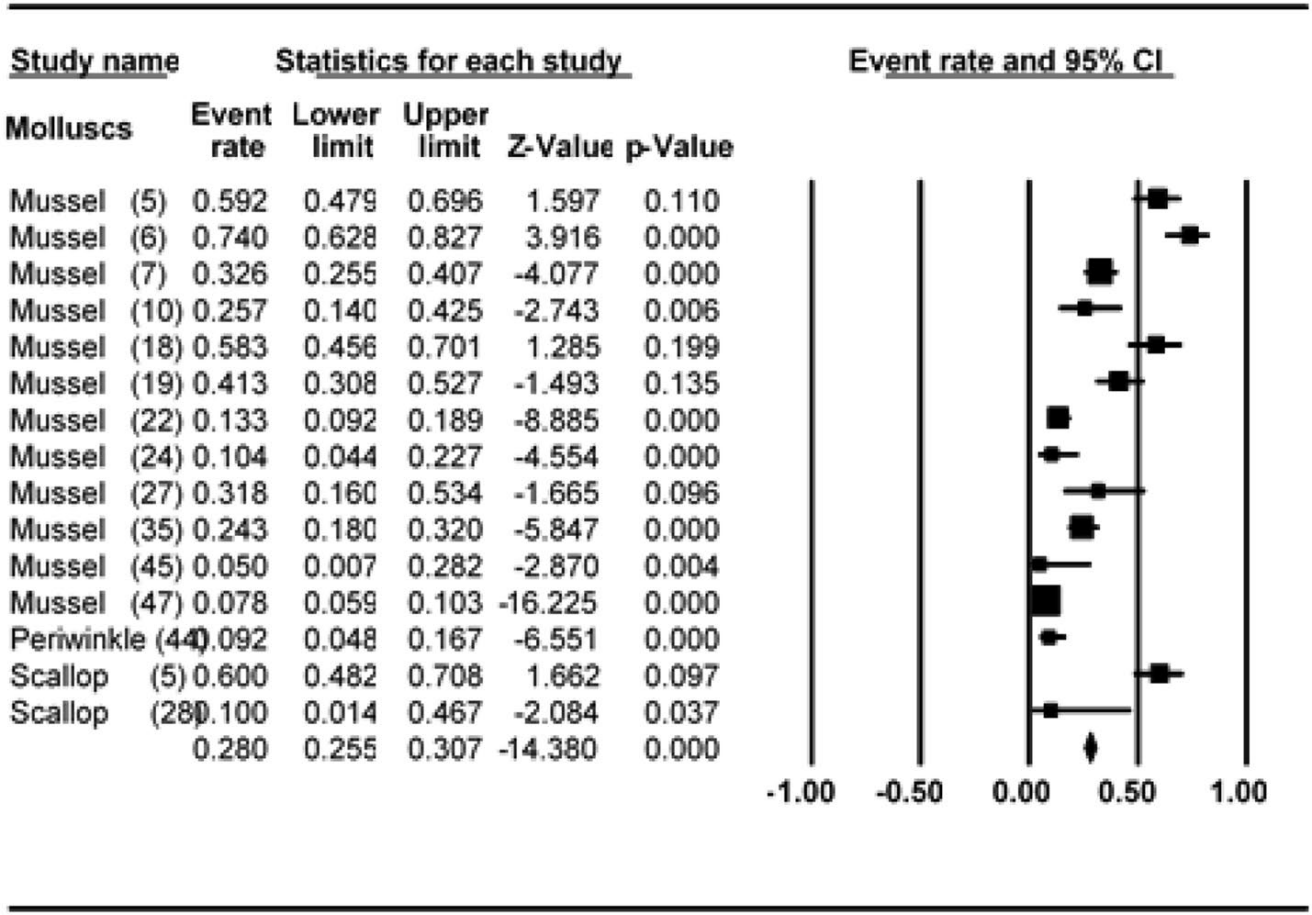
Forest plots of prevalence of *V. parahaemolyticu*s in mussel, scallop and periwinkle for fixed effects meta-analyses. (*Squares* represent effect estimates of individual studies with their 95 % confidence intervals of prevalence with size of *squares* proportional to the weight assigned to the study in the meta-analysis)

**Fig. 3:**
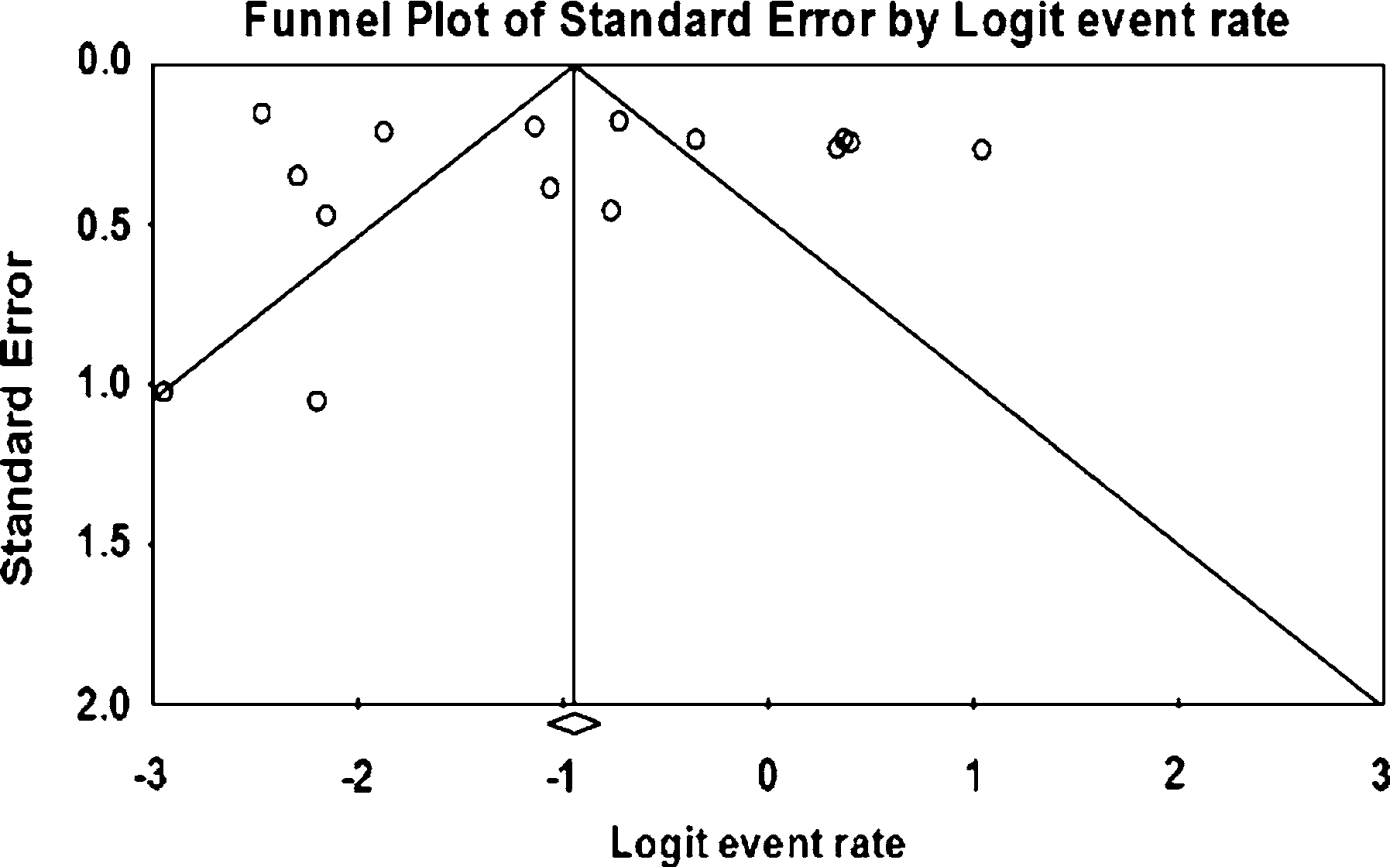
Funnel plot of prevalence of *V. parahaemolyticu*s in mussel, scallop and periwinkle. *Solid vertical line* represents the summary prevalence rate derived from fixed-effect meta-analysis while the *diagonal lines* represent 95 % confidence interval

Meta-analysis of incidence and prevalence of *V. parahaemolyticus* in mussel, scallop, and periwinkle was carried out using data of 1670 samples from 15 studies. The results of estimates of prevalence are summarised in Table 2. The pooled prevalence estimate of *V. parahaemolyticus* was found to be 28.0 % (95 % CI 0.255–0.307) as shown in Table 2. The studies included in this meta-analysis were found to be of significant heterogeneity (Q = 297.293, df = 14, p < 0.001) between 15 studies. Heterogeneity quantified by *I*^2^ index was observed as 95.291 % as shown in the forest plot in Fig. 2. Squares represent effect estimates of individual studies with their 95 % confidence intervals of prevalence with size of squares proportional to the weight assigned to the study in the meta-analysis (Fig. 3).

**Table 2:** Prevalence and meta-analysis statistics of *V. parahaemolyticus* in seafood investigated in the primary studies

df	Sample	Effect size 95 % CI	Heterogeneity			Standard error	Variance
			Q value	p value	*I* ^2^		
14	Mussel, scallop, and periwinkle	28.0 (0.255–0.307)	297.293	0.0000	95.291	0.660	0.436
22	Shrimp, prawn and crab	48.3 (0.454–0.512)	232.099	0.2590	90.521	0.484	0.2345
19	Fish, squid and cephalopod	51.0 (0.476–0.544)	159.368	0.557	88.078	0.460	0.212
17	Clam and cockle	52.9 (0.490–0.568)	132.490	0.145	87.169	0.429	0.184
16	Oyster	63.4 (0.592–0.674)	178.260	0.0000	91.024	0.765	0.586

Q, Cochran’s test; *I* ^2^, inverse variance index; df, degree of freedom

### Meta-analysis of prevalence of *V. parahaemolyticus* in shrimp, prawn and crab

**Fig. 4:**
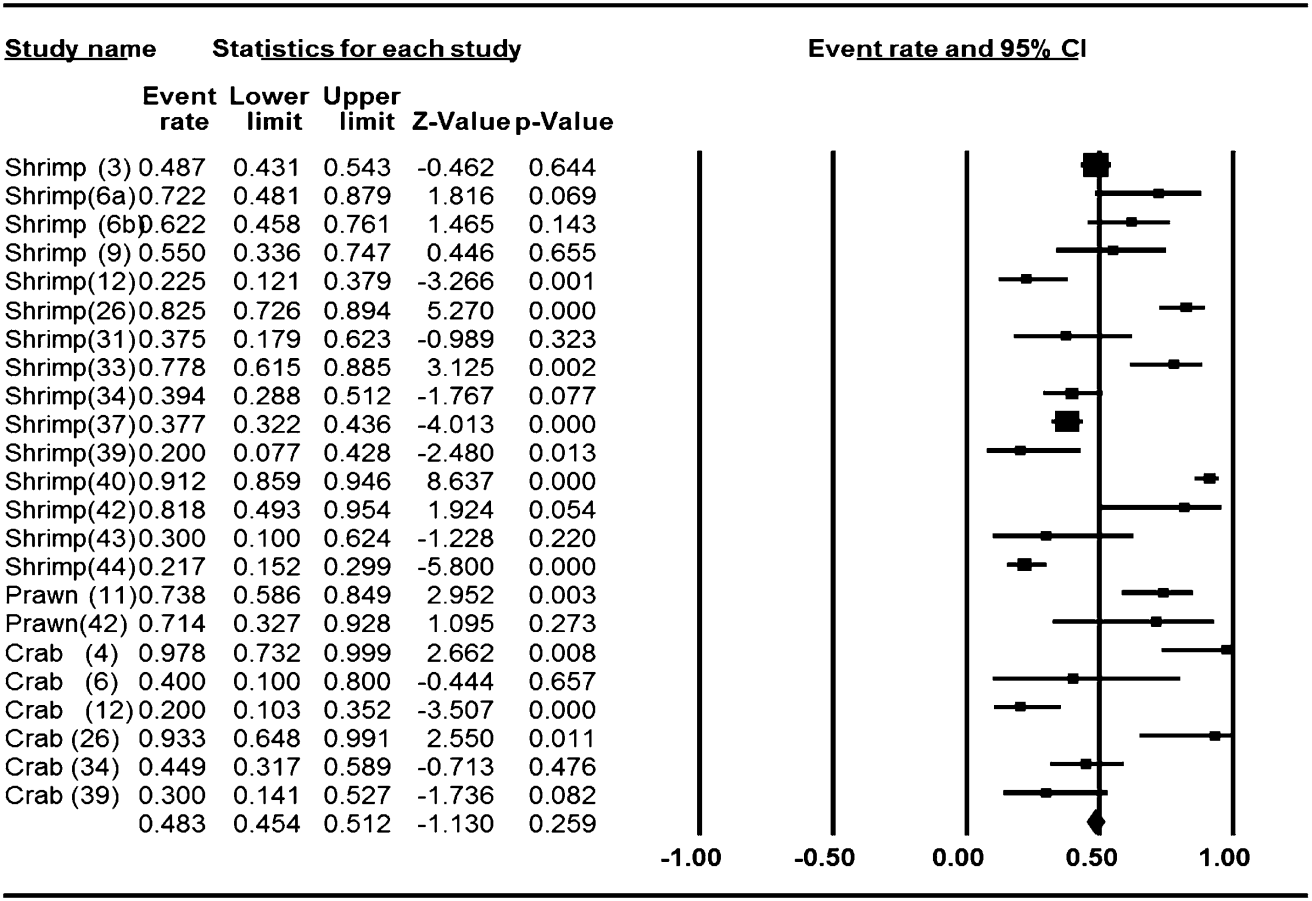
Forest plots of prevalence of *V. parahaemolyticu*s in shrimp, prawn and crab for fixed effects meta-analyses. (*Squares* represent effect estimates of individual studies with their 95 % confidence intervals of prevalence with size of *squares* proportional to the weight assigned to the study in the meta-analysis)

**Fig. 5:**
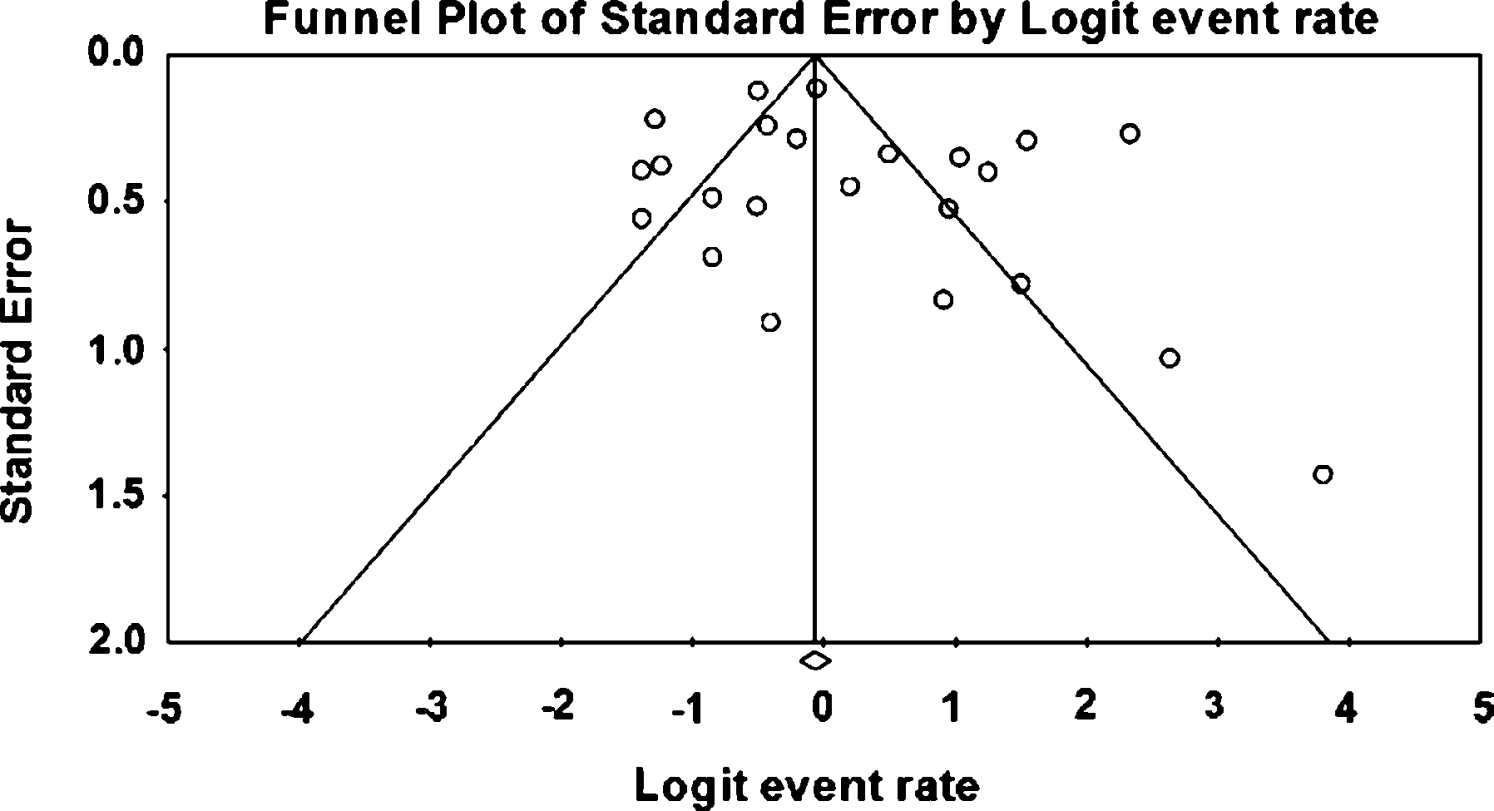
Funnel plot of prevalence of *V. parahaemolyticu*s in shrimp, prawn and crab. *Solid vertical line* represents the summary prevalence rate derived from fixed-effect meta-analysis while the *diagonal lines* represent 95 % confidence interval

Meta-analysis of incidence and prevalence of *V. parahaemolyticus* in shrimp, prawn and crab was carried out using data of 1422 samples from 24 studies. The pooled prevalence estimate of *V. parahaemolyticus* was found to be 48.3 % (95 % CI 0.454–0.512). The primary studies included in this meta-analysis were found to be of significant heterogeneity (Q = 232.099, df = 22, p > 0.001) between 24 studies. Heterogeneity quantified by *I*^2^ index was observed as 90.521 % as shown in the forest plot in Fig. 4. Squares represent effect estimates of individual studies with their 95 % confidence intervals of prevalence with size of squares proportional to the weight assigned to the study in the meta-analysis (Fig. 5).

### Meta-analysis of prevalence of *V. parahaemolyticus* in fish, squid and cephalopod

**Fig. 6:**
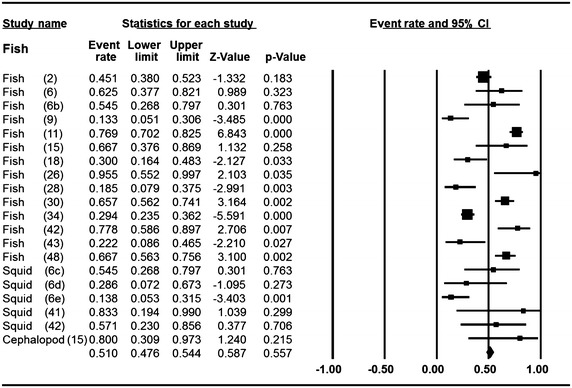
Forest plots of prevalence of *V. parahaemolyticu*s in fish, squid and cephalopod for fixed effects meta-analyses. (*Squares* represent effect estimates of individual studies with their 95 % confidence intervals of prevalence with size of *squares* proportional to the weight assigned to the study in the meta-analysis)

**Fig. 7:**
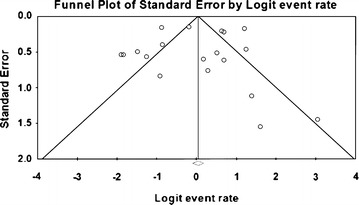
Funnel plot of prevalence of *V. parahaemolyticu*s in fish, squid and cephalopod. *Solid vertical line* represents the summary prevalence rate derived from fixed-effect meta-analysis while the *diagonal lines* represent 95 % confidence interval

Meta-analysis of incidence and prevalence of *V. parahaemolyticus* in fish, squid and cephalopod was carried out using data of 998 samples from 20 studies. The pooled prevalence estimate of *V. parahaemolyticus* was found to be 51.0 % (95 % CI 0.476–0.544). The studies included in this meta-analysis were has found to be significant heterogeneity (Q = 159.368, df = 19, p > 0.001) between 20 studies. Heterogeneity quantified by *I*^2^ index was observed as 88.078 % as shown in the forest plot in Fig. 6. Squares represent effect estimates of individual studies with their 95 % confidence intervals of prevalence with size of squares proportional to the weight assigned to the study in the meta-analysis (Fig. 7).

### Meta-analysis of prevalence of *V. parahaemolyticus* in clam and cockle

**Fig. 8:**
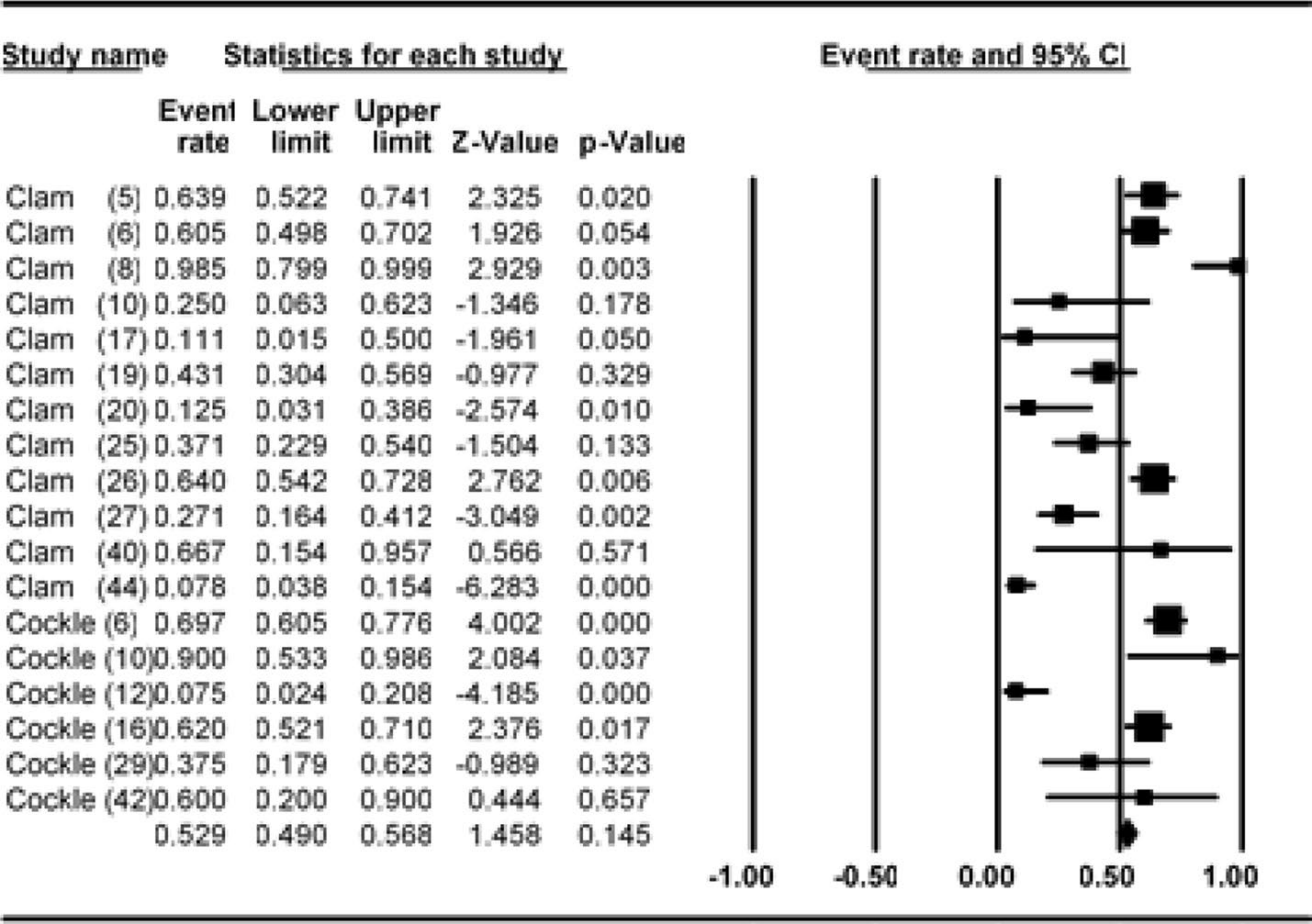
Forest plots of prevalence of *V. parahaemolyticu*s in clam and cockle for fixed effects meta-analyses. (*Squares* represent effect estimates of individual studies with their 95 % confidence intervals of prevalence with size of *squares* proportional to the weight assigned to the study in the meta-analysis)

**Fig. 9:**
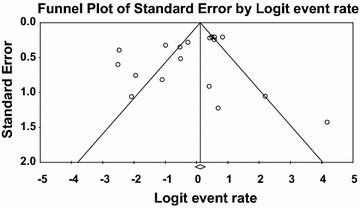
Funnel plot of prevalence of *V. parahaemolyticu*s in clam and cockle. *Solid vertical line* represents the summary prevalence rate derived from fixed-effect meta-analysis while the *diagonal lines* represent 95 % confidence interval

Meta-analysis of incidence and prevalence of *V. parahaemolyticus* in clam and cockle was carried out using data of 830 samples from 18 studies. The pooled prevalence estimate of *V. parahaemolyticus* was found to be 52.9 % (95 % CI 0.490–0.568). The studies included in this meta-analysis were has found to be significant heterogeneity (Q = 132.490, df = 17, p > 0.001) between 18 studies. Heterogeneity quantified by *I*^2^ index was observed as 87.169 % as shown in the forest plot in Fig. 8. Squares represent effect estimates of individual studies with their 95 % confidence intervals of prevalence with size of squares proportional to the weight assigned to the study in the meta-analysis (Fig. 9).

### Meta-analysis of prevalence of *V. parahaemolyticus* in oyster

**Fig. 10:**
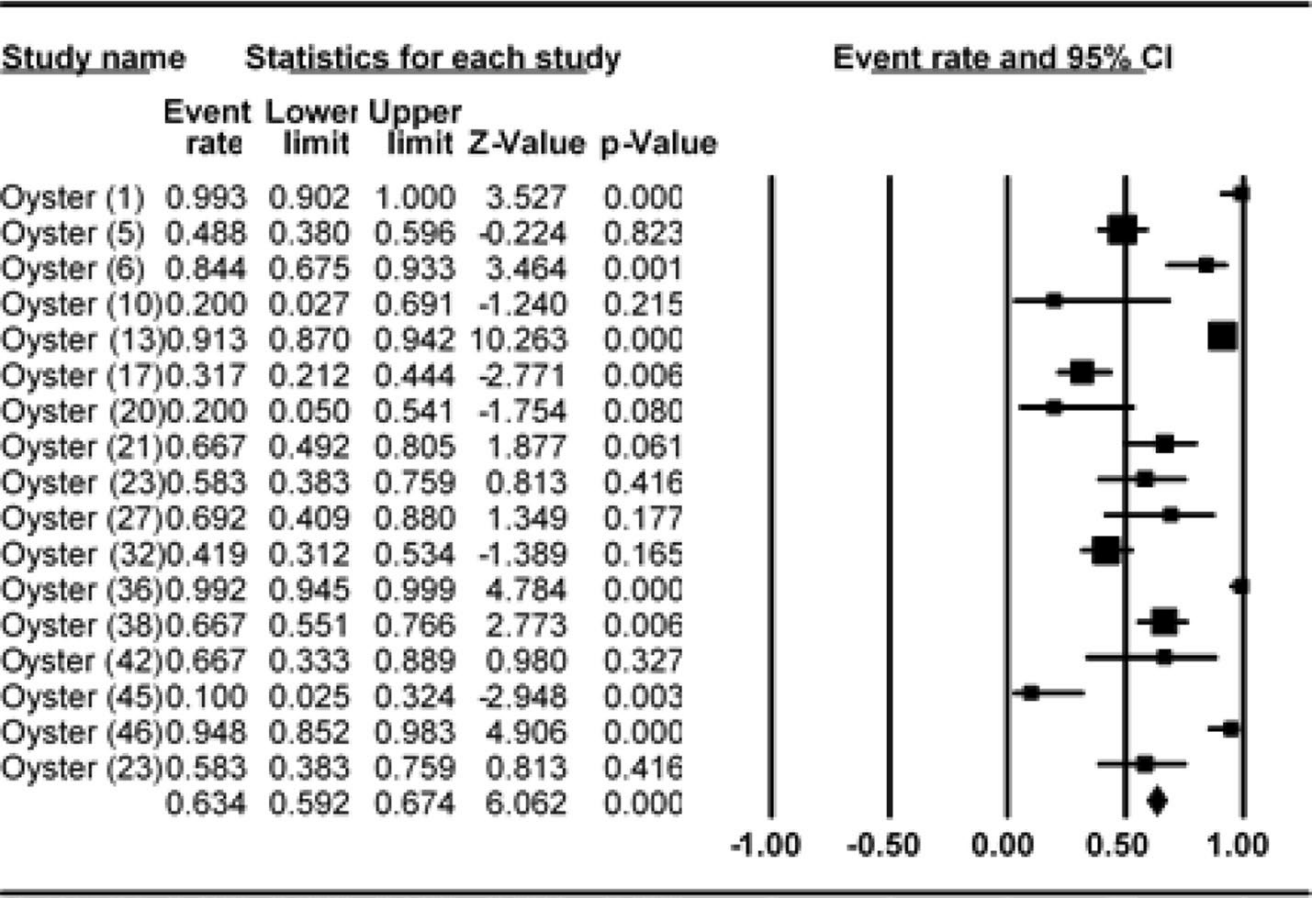
Forest plots of prevalence of *V. parahaemolyticu*s in oyster for fixed effects meta-analyses. (*Squares* represent effect estimates of individual studies with their 95 % confidence intervals of prevalence with size of *squares* proportional to the weight assigned to the study in the meta-analysis)

**Fig. 11:**
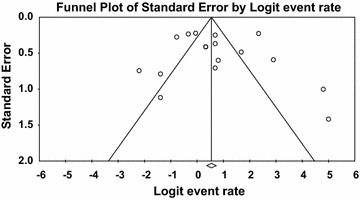
Funnel plot of prevalence of *V. parahaemolyticu*s in oyster. *Solid vertical line* represents the summary prevalence rate derived from fixed-effect meta-analysis while the *diagonal lines* represent 95 % confidence interval

Meta-analysis of incidence and prevalence of *V. parahaemolyticus* in oyster was carried out using data of 951 samples from 17 studies. The pooled prevalence estimate of *V. parahaemolyticus* was found to be 63.40 % (95 % CI 0.592–0.674). The studies included in this meta-analysis were has found to be significant heterogeneity (Q = 178.260, df = 16, p < 0.001) between 17 studies. Heterogeneity quantified by *I*^2^ index was observed as 91.024 % as shown in the forest plot in Fig. 10. Squares represent effect estimates of individual studies with their 95 % confidence intervals of prevalence with size of squares proportional to the weight assigned to the study in the meta-analysis (Fig. 11).

### Publication bias among the primary studies

Both publication bias and quality of primary studies are limiting factors in any meta-analytical study (Noble Jr. 2006). In meta-analysis, publication bias is usually graphically assessed using funnel plot (Soon et al. 2012; Gonzales-Barron and Butler 2011). This was obtained by plotting of study size (usually standard error or precision) on the vertical axis as a function of effect size on the horizontal axis. In this current study, publication bias could be observed among the primary studies due to asymmetric nature of the plots. Solid vertical line in the funnel plots represents the summary of prevalence rate derived from fixed-effect meta-analysis while the diagonal lines represent 95 % confidence interval. Studies with large samples appeared toward the top of the graph, and tend to cluster near the mean effect size while studies with smaller samples appeared toward the bottom of the graph. It should be noted that sampling variation in effect size estimates in the studies with smaller seafood samples affects the plots.

## Conclusion

In conclusion, higher prevalence rate of *V. parahaemolyticus* was observed in oysters than other seafood investigated. The occurrence and prevalence of *V. parahaemolyticus* is of public health importance, hence, more studies involving seafood such as mussels need to be investigated. Additionally, the study is a trial to develop a new data analysis tool. There is need to investigate prevalence of this pathogen in other seafood and also intervention strategies to reduce *V. parahaemolyticus* in seafood.
